# Using Mobile Fruit Vendors to Increase Access to Fresh Fruit and Vegetables for Schoolchildren

**DOI:** 10.5888/pcd9.110222

**Published:** 2012-05-24

**Authors:** June M. Tester, Irene H. Yen, Barbara Laraia

**Affiliations:** Author Affiliations: Irene H. Yen, Barbara Laraia, University of California, San Francisco, California.

## Abstract

This study explored the extent to which schoolchildren purchased precut and bagged fruits and vegetables from a mobile fruit vendor (*frutero*). During 14 days in fall 2008, a *frutero* sold fruits and vegetables at the entrance of an elementary school; 59% of the *frutero*’s 233 consumers of 248 items were elementary-school students. With each successive day, an average of 1 additional bag of fruits and vegetables was sold by the *frutero* and 1.5 fewer nonnutritious foods by a competing vendor. Policies encouraging the sale of nutritious foods from mobile food vendors may increase access for schoolchildren.

## Objective

Mobile food vendors are sometimes found in Hispanic and African American neighborhoods, often where there are few large food outlets (1,2). Since 2008, the Green Carts program in New York City has provided incentives to vendors to sell whole fruits and vegetables in areas that have limited access to such food (3). There is value in exploring public health strategies that encourage the sale of nutritious food by mobile vendors, including such approaches as broadening the zones and locations in which vendors are permitted to sell (4,5).

In Oakland, California, mobile vendors known as *fruteros* sell fresh, precut, and bagged fruit (6-9). Since 2001, these vendors have complied with city regulations and health codes requiring a central kitchen (9). However, local regulations prohibit mobile food vendors — including these *fruteros* — from selling near schools and parks (10). Such regulations exist in other cities such as Phoenix, Arizona; San Antonio, Texas; San Diego, California; and San Jose, California (4). We are unaware of any school or city policies that encourage improved food access for schoolchildren through mobile vendors.

The objective of this study was to examine the extent to which schoolchildren purchased fruits and vegetables from a *frutero* after school. Secondary objectives were to compare *frutero* sales with those of competing vendors and examine the effect of the *frutero* on the greater mobile vending environment.

## Methods

We conducted this pilot intervention outside an elementary school campus (kindergarten through 5th grade) in Oakland, California. During the 2008–2009 academic year, there were 279 students; 41% were Hispanic, 33% were African American, 15% were Asian, less than 1% were white, and 74% were eligible for free or reduced price lunch (11).

### Intervention

In October 2008, we obtained school permission for a single *frutero* to sell fruits (eg, mango) and vegetables (eg, jicama) at the entrance to the school property at the close of each school day. The *frutero* sold products that had been precut and packaged in a central kitchen into snack-sized bags holding a one-half–cup serving. Bags were chilled with ice and sold for $1.50. We based the design of this intervention on observations of sales at *fruteros* (2) and with input from the *frutero* vendor’s association. Neither the school nor the teachers actively promoted this intervention among students. This study was approved by the institutional review board of Children’s Hospital & Research Center Oakland.

### Observations of consumers and transactions

Using methods described elsewhere (2), research staff counted consumers at the *frutero* and at competing vendors within 1 block of the *frutero*. They characterized consumers according to best guess of race/ethnicity and age and categorized them as preschoolers, elementary school students, teenagers, or adults. They also counted the number of transactions and characterized the type and cost of each item sold at each vendor.

Each afternoon for 19 days (5 baseline days, 14 intervention days), at least 3 research staff simultaneously observed all vendors within 1 block of the school entrance, beginning when the school bell rang and ending whenever the vendor left because sales had dwindled.

### Observations of nonintervention vendors

To determine whether the presence of the *frutero* would affect the greater mobile vending environment, we also counted the number of (nonintervention) vendors within a quarter-mile of the school entrance (not including the competing vendors within 1 block). We created a one-quarter–mile network buffer around the school using Arc View 9.2 (ESRI, Redlands, California). Pairs of researchers counted all such vendors on 17 days after school (6 days before, 7 days during, and 4 days after the intervention); they did not observe sales.

### Analysis

We conducted linear regression using day of the intervention as a predictor for sales per afternoon of bags of fruits and vegetables at the *frutero* (1–14 d) and for sales at the competing vendors within 1 block of the *frutero* (1–19 d) We used Stata 9.2 (StataCorp LP, College Station, Texas).

## Results

We found 2 types of competing vendors: an ice cream vendor and a cotton candy vendor. At least 1 competing vendor was present for 12 afternoons (Table 1). Of the *frutero*’s 233 consumers, 59% were elementary school students; for 27% of *frutero* transactions, no adults were present (Table 2).

**Table 1:** Items Sold by a *Frutero* and Competing Vendors Within a Block of an Elementary School, Oakland, California, 2008

Day	*Frutero*	Ice Cream Vendor	Cotton Candy Vendor
**Baseline**			
Day 1	NP	19	NP
Day 2	NP	15	3
Day 3	NP	44	NP
Day 4	NP	32	NP
Day 5	NP	39	NP
**Intervention**			
Day 6	14	NP	NP
Day 7	9	35	NP
Day 8	4	18	NP
Day 9	17	24	1
Day 10	17	NP	NP
Day 11	15	NP	NP
Day 12	28	2	NP
Day 13	21	36	NP
Day 14	15	NP	NP
Day 15	23	NP	NP
Day 16	23	7	NP
Day 17	20	NP	NP
Day 18	21	NP	NP
Day 19	21	17	NP

Abbreviations: NP, not present.

**Table 2:** Transactions, Consumers, and Items Sold by a *Frutero* and Competing Vendors Within a Block of an Elementary School, Oakland, California, 2008

Variable	*Frutero*	Competing Vendors
**Transactions**		
Mean duration of sales (per vendor) per afternoon, min	26	16
Mean number of vendors per afternoon	1	1.1
Total transactions, n	193	212
Transactions with children only, n (%)	52 (27)	76 (36)
Transactions with adults only, n (%)	44 (23)	21 (10)
Transactions with children and adults, n (%)	97 (50)	115 (54)
**Consumers**		
Total, n	233	266
Female, n (%)	151 (65)	144 (54)
Male, n (%)	82 (35)	122 (46)
**Age group, n (%)**		
Preschooler	10 (4)	14 (5)
Elementary school student	138 (59)	193 (73)
Teenager	9 (4)	7 (3)
Adult	74 (32)	48 (18)
Observer unsure of age group	2 (1)	4 (1)
**Race/ethnicity of elementary school students, ^a^ n (%)**		
Hispanic	75 (54)	113 (59)
African American	50 (36)	44 (23)
Asian	13 (9)	2 (1)
White	0	24 (12)
Observer unsure of race/ethnicity	0	10 (5)
**Items sold**		
Total, n	248	292
Items sold per afternoon, mean (range), n	18 (4–28)	24 (2–44)
Cost per item, mean (SD), $	1.50 (0)	1.13 (0.24)
Items sold per minute, n	0.8	1.5

Abbreviation: SD, standard deviation.

^a^ The race/ethnicity of the school population was 41% Hispanic, 33% African American, 15% Asian, and <1% white.

The *frutero* sold an average of 17.7 bags of fruits and vegetables each afternoon during an average of 26 minutes of sales, and for the last 5 days of observation, at least 20 bags were sold each afternoon. Overall, the *frutero* sold 248 bags during 324 minutes of observed sales (an approximate rate of 0.8 bags per minute) (Table 1). A β coefficient of 0.95 (*P* < .001) for *frutero* sales suggests 1 additional bag was sold on each successive day; a β coefficient of −1.48 (*P* = .02) for sales of competing vendors suggests 1.5 fewer nonnutritious items were sold on each successive day.

We completed quarter-mile buffer observations in an average of 12.8 minutes. The mean number of nonintervention vendors decreased: 2.3 (range, 0–4; standard deviation [SD], 1.3) before, 1.4 (range, 0–3; SD, 1.1) during, and 0.8 (range, 0–2; SD, 0.9) after the intervention.

## Conclusion

*Fruteros* are found in neighborhoods that have a high density of Hispanic immigrants; children and adults buy precut fruits and vegetables from these vendors even in the presence of other vendors selling nonnutritious options (2). Students in our study also made purchases on their own. The number items sold by *fruteros* per minute in our study was higher than that previously observed in the community (0.4 items per min) (2).

This study has limitations. It was a brief intervention at a single school. We cannot confirm that children purchased fruits and vegetables instead of high-calorie energy-dense snacks; future research on how “healthy” mobile vendors affect overall consumption is warranted. By study end, we found fewer competing vendors, perhaps because of competition from the *frutero,* who had a prime location, or perhaps because the other vendors, who are not permitted to sell near the school, were concerned about scrutiny.

We demonstrated the feasibility of a sanctioned vendor to sell nutritious food items after school and suggest that the presence of this vendor may decrease sales at vendors selling less healthful items. Interventions like ours have the potential to increase access to healthful food to children in the after-school environment.
